# Mechanisms Underlying the Osteo- and Adipo-Differentiation of Human Mesenchymal Stem Cells

**DOI:** 10.1100/2012/793823

**Published:** 2012-03-12

**Authors:** Yu Zhang, Dilaware Khan, Julia Delling, Edda Tobiasch

**Affiliations:** Department of Natural Sciences, Bonn-Rhine-Sieg University of Applied Sciences, von-Liebig-Straße 20, 53359 Rheinbach, Germany

## Abstract

Human mesenchymal stem cells (hMSCs) are considered a promising cell source for regenerative medicine, because they have the potential to differentiate into a variety of lineages among which the mesoderm-derived lineages such adipo- or osteogenesis are investigated best. Human MSCs can be harvested in reasonable to large amounts from several parts of the patient's body and due to this possible autologous origin, allorecognition can be avoided. In addition, even in allogenic origin-derived donor cells, hMSCs generate a local immunosuppressive microenvironment, causing only a weak immune reaction. There is an increasing need for bone replacement in patients from all ages, due to a variety of reasons such as a new recreational behavior in young adults or age-related diseases. Adipogenic differentiation is another interesting lineage, because fat tissue is considered to be a major factor triggering atherosclerosis that ultimately leads to cardiovascular diseases, the main cause of death in industrialized countries. However, understanding the differentiation process in detail is obligatory to achieve a tight control of the process for future clinical applications to avoid undesired side effects. In this review, the current findings for adipo- and osteo-differentiation are summarized together with a brief statement on first clinical trials.

## 1. Stem Cells

Stem cells are defined as a type of pluripotent or multipotent cells, which have two typical features: self-renewal and have the potential to differentiate into several different cell lineages. According to the source they are obtained from, stem cells can be classified into embryonic stem cells (ESCs) which are isolated from the inner cell mass of blastocysts and adult stem cells found in various tissues of the mature organism. Adult stem cells are divided mainly into hematopoietic stem cells (HSCs) and mesenchymal stem cells (MSCs), but various other stem- and precursor cells have been found in a variety of different organs or tissues in the last years such as neural stem cells or skin stem cells [1–4]. Compared to ESCs that have the potency to differentiate into all kinds of cells lineages, these adult stem cells can only differentiate into several lineages. For instance, HSCs can differentiate into the cells blood which is composed of monocytes, neutrophils, lymphocytes, erythrocytes, and platelets [5]. More recently, a new and interesting source of stem cells has been created by Takahashi and Yamanaka named induced pluripotent stem cells (iPS) [6]. iPS are obtained by genetic reprogramming of differentiated somatic cells of adult tissue using pluripotent factors like Oct4, Sox2, Klf4, and c-myc [7, 8] or Oct4, Sox2, Lin28, and Nanog [9]. Depending on the tissue source, they can even be generated by only one factor [10, 11]. They are regarded as a promising stem cell source for future repair of tissues or organs, especially since they are ethically not problematic. However, together with ESCs, iPS can easily form teratomas [12] and seem to be immunogenetically active after transplantation into the host [13]. In contrast, MSCs show some unique features: they are immunosuppressive and immunoprivileged [14]. They also show no detectable teratoma formation, high migration and motility and further display feasibility and safety use *in vivo* in clinic trials so far [15–18].

## 2. Multilineage Differentiation Potential of Mesenchymal Stem Cells

### 2.1. The Mesenchymal Stem Cell Character

**Figure 1:**
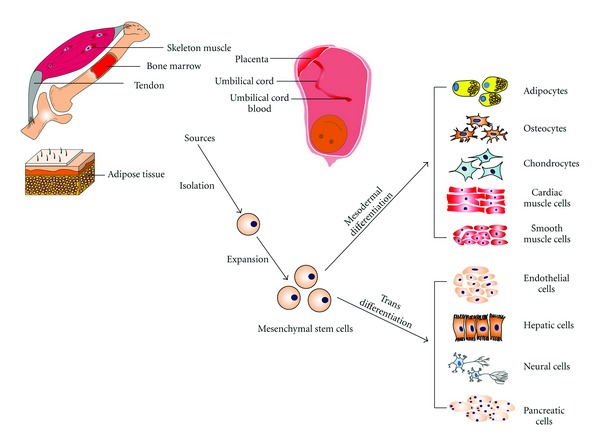
Multipotent Differentiation of Mesenchymal Stem Cells from Different Sources. MSCs can be obtained from skeleton muscle, bone marrow, tendon, adipose tissue, placenta, umbilical cord, and umbilical cord blood. MSCs have the potential to generate mesodermal lineages such as adipocytes, osteoblasts, and chondrocytes; on the other hand, they can also trans-differentiate into some cells lineages from other germ layers such as adipocytes, osteocytes, chondrocytes, cardiac muscle cells smooth muscle cells, and endothelial cells form mesodermal layer, neural cells from ectodermal layer, and hepatic cells and pancreatic cells from endodermal layer.

MSCs are adult stem cells, which can self-renew and stay in the “undifferentiated” state due to some intrinsic or extrinsic suppressed factors until activated [19–22]. MSCs have been originally isolated from bone marrow (BM) [23, 24], and later similar populations were successfully harvested from other adult tissues such as adipose tissue (AT), tendon, peripheral blood (PB), skeleton muscle (SM), and recently from the trabecular bone [25–28]. At the same time, they can be generated from some neonatal tissues, umbilical cord (UC), umbilical cord blood (UCB), and particular parts of the placenta [29, 30] (see Figure 1). Although MSCs derived from bone marrow (BM-MSC) were first obtained and largely studied and used, MSC generated from AT (AT-MSC), PB (PB-MSC), and UCB (UCB-MSC) do not need an invasive procedures for isolation compared to BM-derived cells [31]. However, with the rising number of isolation procedures and the use of different sources, there is an important and urgent issue that need to be solved to compare the outcomes: the definition of an international standard for the MSC character [32]. In 2006, the International Society of Cellular Therapy (ISCT) proposed minimal criteria for defining mesenchymal stem cells: these cells can adhere to the plastic under standard culture condition. They positively express CD73 (SH2), CD90, and CD105 (SH3) and negatively express CD34, CD45, CD14 or CD11b, CD79*α*, or CD19 and HLA-DR. In addition, they should have the multipotency to differentiate into osteoblasts, adipocytes, and chondrocytes* in vitro* as demonstrated by specific stainings [33].

### 2.2. Source Difference and Comparison of the MSCs from Different Sources

MSCs derived from different sources pose same or similar features. However, many publications have reported that they have some differences in their proliferation rates, surface marker expressions, multipotency, and some other specific markers. This might be used to find the best source of MSCs to address specific qualities for replacement strategies in regenerative medicine [34, 35]. Generally, MSCs obtained from neonatal tissues have the significant advantage of avoiding invasive procedures which are usually accompanied with infection risk if compared to those from adult tissues [31]. They also show higher expansion and engraftment capacities compared to MSCs derived from BM [36, 37]. On the other hand, the success rate of isolation was only 63% from UCB if compared to those derived from BM and AT. Interestingly, MSCs derived from UCB seem not to have the capacity to differentiate towards the adipogenic lineage [38, 39]. The proliferation capacity was higher in UC-MSCs than in BM-MSCs, and contact inhibition was observed in BM-MSCs but not in UC-MSCs [40, 41]. MSCs derived from cartilage exhibited the highest resistance against hydrogen peroxide-induced apoptosis, and AT-MSCs pose the highest proliferation rate and tolerance to serum deprivation-induced apoptosis [42]. The doubling time of population of AT-MSCs is 3/4 of BM-MSCs. AT-MSCs also have different doubling times if derived from different regions [43, 44]. For example, AT-MSCs derived from omental regions proliferated slower than those from subcutaneous region [45]. MSCs from BM and placenta were shown to have a higher migration capacity than those from UC. This seemed to be regulated by increased expression of cathepsin B, cathepsin D, prohibitin and decreased expression of plasminogen activator inhibitor-1 (PAI-1), and manganese superoxide dismutase [46]. UCB-MSCs need shorter time to differentiate into osteogenic lineages than BM-MSCs [47]. Compared to adult-tissue-derived MSCs, neonatal MSCs also have a stronger immunosuppressive capacity and show lower immunogenicity. Therefore, they seemed to be a very reasonable source for therapeutic applications [48].

BM-MSC and AT-MSC are the two most frequently investigated MSCs. AT-MSCs can be obtained as a population of 5000 cells from one gram adipose tissue compared to only about 0.01% cells isolated from the interface after density gradient centrifugation of bone marrow aspirates. This means 500 times more cells can be obtained from AT than from equal amounts of BM [3, 49]. No remarkable difference in their morphology and immune phenotype was observed in BM-MSC and AT-MSCs [50]. But later Peng and colleagues reported that BM-MSCs are larger than AT-MSC [42], and that their proliferative activity is higher than BM-MSCs [42, 51]. Some surface antigen expressions differ in BM-MSC and AT-MSC: CD49d, CD54, CD34, and CD106. CD49d was expressed only in AT-MSC, and the expression of CD106 was detected only in BM-MSCs [26, 52]. The expression of chemokine receptors such as CCR1, CCR7, CXCR4, CXCR6 was increased in AT-MSCs compared to BM-MSCs [53]. By using a human genome microarray, 25 genes were predominantly up-regulated in BM-MSCs, AT-MSCs and UCB-MSCs compared to fibroblasts [54]. Additionally, they found that the mesoderm-specific transcript homolog (MEST) is expressed highest in BM-MSC and the connective tissue growth factor (CTGF), and the BMP antagonist 1 expressions are highest in UCB-MSC. At the same time, cyclin B2 (CCNB2), cell division cycle associated 8 (CDCA8), and Ki-67 were higher expressed in AT-MSC, which indicates that AT-MSC might have the highest proliferative capacity. This result may explain the above findings that AT-MSCs multiplied faster than BM-MSCs [42, 51]. Meanwhile cell population, maximal life span, and multipotential of BM-MSC decrease with increasing the donor's age [55, 56]. These differences of MSCs (summarized in Table 1) could be due to the region they are derived from or due to different isolated methods. In summary, MSCs isolated from adipose tissue can express all typical markers, simultaneously be isolated in large amount without additional pain or highly invasive procedures, and show strong apoptosis tolerance. Therefore, they are used and investigated as important and promising stem cells for regenerative medicine [57].

### 2.3. Mesodermal Differentiation and Transdifferentiation

Due to mainly mesodermal origin derived, mesenchymal stem cells always were regarded as an attractive source for differentiating into cells of this source such as osteoblasts (bone), adipocytes (fat tissue), and chondrocytes (cartilage) [35]. Recently some publications reported that MSCs also have the potential to differentiate into several additional cell lineages from the mesoderm. For example, MSCs derived from different regions showed myogenic potential and can differentiate into muscle lineages like skeleton muscle cells [58–60], smooth muscle cells [61–63], and cardiac muscle cells [64]. Interestingly, MSCs can differentiate into cells derived from other germ layers as well (see Figure 1). For example, MSCs can differentiate into neuron-like cells *in vitro* [65, 66], and into astrocytes and neurons after implanting into the mouse brain *in vivo *[67]. Hepatocyte-like cells can also be generated from MSCs* in vitro* and *in vivo* [68, 69]. MSCs pose the ability to differentiate into endothelial cells too [61, 70]. They have also been considered a good source for insulin producing cells, which could be applied for diabetes therapy [71, 72]. The reason for this might be that although MSCs are originated from mesoderm, they have parts which are originated from other germ layers as well. Other authors describe this phenomenon as “transdifferentiation”, meaning that stem cells differentiate into cells from another germinal layer the stem cells are derived from. Transdifferentiation, although not applicable to mesenchymal stem cells, is a widely used term often to doubt a specific differentiation since lineage commitment is thought to be not reversible until iPS came up [73]. On the other hand, the physical properties of the scaffold can also determine MSC differentiation [74]. For example, MSCs cultured in stiff scaffolds are easily differentiated into osteoblasts, and with the decrease of elasticity, MSCs showed the potential of myogenic, adipogenic and neurogenic differentiation, respectively [75]. To make a long story short, MSC fate is influenced by their environment, including growth factors, mechanical or physical stimulation, cell-cell attachment or interactions, and cell density [76]. However, this multipotency of MSCs might also be due to another reason, which is also postulated for UCB and wildly discussed. MSCs might not be pure and specific adult stem cells, but instead they might be a diverse mixture of many specific lineage progenitor cells [77].

### 2.4. Osteogenic Differentiation of MSCs

Multipotent stem cells such as MSCs express markers of multiple cell lineages [78]. These markers keep the cells in an undifferentiated state through negative feedback mechanisms. To commit the cells towards a specific lineage cytokines* in vivo* and induction factors *in vitro* is necessary. To induce *in vitro* osteogenesis of MSCs, combinations of different induction factors have been suggested (Table 2). In addition to supplements added to the basal medium, other techniques to optimize osteogenic induction have been investigated as well. In some studies, mechanical stress [79], pulsed electromagnetic field (PEMF) [80], and hydrostatic pressure (HP) [81] were added to the osteogenic factors, while in others these factors were used to stimulate osteogenic differentiation without osteogenic induction supplements.

Dexamethasone is a potent stimulator of *in vitro* osteogenesis and induces the expression of the runt-related transcription factor 2 (Runx2), Osterix (Osx), and bone matrix proteins [82]. Ascorbic acid and *β*-glycerophosphate increase type I collagen secretion [83].

Jansen and colleagues cultured BMSCs in osteogenic medium and treated them with a pulsed electromagnetic field (PEMF). PEMF treatment increased the intensity of osteogenic differentiation [80, 84]. PEMF has been suggested to enhance DNA synthesis through which it affects *in vitro* proliferation and differentiation of bone cells [85, 86]. During differentiation, it increases the bone marker gene expressions and also promotes calcified matrix production [87].

To investigate the effect of extracellular matrix (ECM) proteins on osteogenic differentiation of hMSCs, Salasznyk and coworkers coated tissue culture plates with repetitive collagen I and collagen IV, laminin-I, and vitronectin. These ECM proteins were found in bone marrow. This study showed that culturing of hMSCs on purified vitronectin and collagen I without osteogenic medium was sufficient to induce osteogenic differentiation [88]. Collagen I has been suggested to induce calcification of the stromal cell matrix [89]. Both, collagen type I and vitronectin have been reported to promote osteogenic differentiation of MSCs [90]. 

Eslaminejad and colleagues coated plastic surfaces of culture plates with matrigel. Matrigel is composed of laminin, collagen IV, proteoglycan, heparin sulfate, entactin, nidogen, and growth factors like transforming growth factor beta (TGF-beta), epidermal growth factor (EGF), insulin-like growth factor 1, bovine fibroblast growth factor (bFGF), and platelet-derived growth factor (PDGF) [91]. These factors create a microenvironment that regulates the proliferation and differentiation of hMSCs. HMSCs were cultured on matrigel-coated and plastic surface and induced towards the osteogenic lineage. It has been reported that hMSCs on matrigel-coated culture plates showed significantly stronger osteogenic differentiation if compared to hMSCs on plastic surface [92].

In another study, MSCs were cultured in linear 3D type I collagen matrices and subjected to different uniaxial cyclic tensile strain for 7 or 14 days. The results of this study showed that BM-MSCs in 3D collagen matrices under cyclic strain can differentiate towards osteogenic lineage without the addition of osteogenic supplements [93]. Whereas Yourek and colleagues reported that shear stress stimulates MSCs towards an osteoblastic phenotype in the absence of chemical induction [79].

Hess and coworkers investigated the effect of hydrostatic pressure (HP) stimulation on MSCs seeded on collagen-based artificial extracellular matrices. They coated artificial extracellular matrices generated from collagen and chondroitin sulfate onto polycaprolactone-co-lactide substrates. MSCs were seeded and subjected to cyclic HP at various time points during 21 days to investigate the effects of biochemical, mechanical, and combined biochemical and mechanical stimulations. Both HP and coated artificial matrices containing collagen and chondroitin sulfate promoted the osteogenic differentiation of MSCs individually, and a combination of both showed a synergistic effect on osteogenic induction of MSCs on scaffolds [81].

Sundelacruz and colleagues investigated the effect of a membrane potential on hMSCs differentiation towards the osteogenic lineage. Stem cells show a unique electrophysiological profile during their undifferentiated state [94]. Ionic currents and channels have been found to play a role in stem cell differentiation [95, 96]. Sundelacruz showed that treatment of hMSCs with hyperpolarizing reagents increased the strength of osteogenic differentiation [97].

Taken together, all these studies show that chemical supplements and physical or mechanical factors can induce osteogenic differentiation of MSCs. A combination of these factors can be used to achieve an optimal differentiation potential of MSCs towards the osteogenic lineage.

The commitment and differentiation of MSCs towards osteogenic lineage is regulated by a certain group of factors [98]. Among these factors, the initial and most specific marker is Runx2. Runx2 activates and regulates osteogenic differentiation by two independent signaling pathways via transforming growth factor-beta 1 (TGF *β*1) and bone morphogenetic protein 2 (BMP2) [99, 100].

Along with Runx2, BMP2 and distal-less homeobox 5 (Dlx5) commit MSCs towards the osteogenic lineage. Commitment is the process that restricts MSCs to respond and undergo differentiation towards a specific lineage [101, 102]. In addition to the induction of osteogenic differentiation, Runx2 inhibits the differentiation of MSCs towards the adipogenic lineage [103]. BMP2 induces the expression of Osx independent of Runx2 [104].

**Figure 2:**
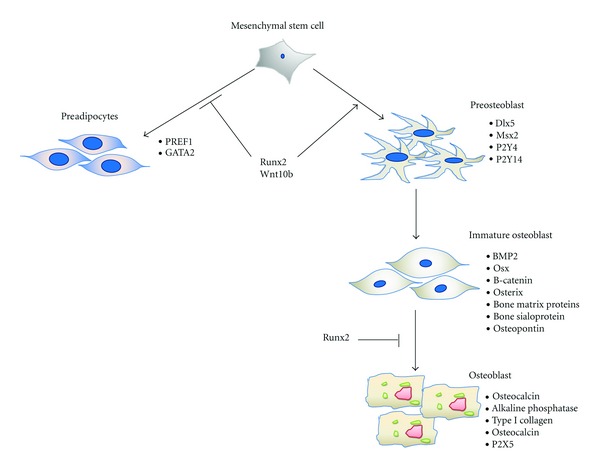
Change and differentiation of mesenchymal stem cells towards osteoblasts. Runx2 commits MSCs towards osteogenic lineage and inhibits adipogenic differentiation. After commitment, MSCs are differentiated into preosteoblasts which express runt-related transcription factor 2 (Runx2), distal-less homeobox 5 (Dlx5), and msh homeobox homologue 2 (Msx2). Preosteoblasts differentiate into immature osteoblasts. Immature osteoblasts express bone morphogenetic protein 2 (BMP2), Osterix (Osx), *β*-catenin, bone matrix proteins, bone sialoprotein, osteopontin and develop into mature osteoblasts. Mature osteoblasts express osteocalcin, alkaline phosphatase, and type I collagen.

Following commitment, MSCs are differentiated into preosteoblasts. Preosteoblast are elliptical in shape with an elongated nucleus and are capable of proliferation (see Figure 2). They express Runx2, D1x5, msh homeobox homologue 2 (Msx2), P2Y4 and P2Y14 [35, 105], and few markers of osteoblasts such as ALP, type I collagen, and osteopontin (OPN), but their expression is weaker than immature osteoblasts. Alkaline phosphatase is one of the early proteins and regulates bone mineralization.

*β*-catenin, Runx2, and Osx differentiate preosteoblasts into immature osteoblasts [106]. These cells are spindle shape (see Figure 2). They express bone matrix protein, bone sialoprotein, and OPN [106].

At later stages, Runx2 inhibits the maturation of osteoblasts [107]. Osx causes the terminal maturation of osteoblasts and induces osteocalcin expression [108]. When osteoblasts are completely differentiated they become cuboidal (see Figure 2) and produce a self-mineralized organic matrix [109]. The Golgi bodies and rough endoplasmic reticulum are well developed in mature osteoblasts as a result of increased need for protein production. The expression of OPN is reduced in mature osteoblasts; while the expression of other proteins such as P2X5 [35], alkaline phosphatase [110], collagen type I [110, 111], and osteocalcin [111] is increased.

### 2.5. Adipogenic Differentiation of Mesenchymal Stem Cells

*In vivo* MSCs presumably receive cytokine signals for differentiation [112], but *in vitro* they cannot get such signals from other cells. Therefore, certain induction factors are needed to induce MSCs towards the adipogenic lineage. To induce *in vitro* adipogenic differentiation of mesenchymal stem cells, three induction factors are required that are dexamethasone, indomethacin, and insulin [26, 113]. Dexamethasone is a synthetic glucocorticoid agonist that acts as a potent-stimulating agent during the differentiation of mesenchymal stem cells [114]. Indomethacin is a nonsteroidal antiinflammatory drug that induces adipogenic differentiation by activating PPAR*γ* [115, 116]. Insulin promotes adipogenesis through at least four known mechanisms [117]. Insulin triggers adipogenesis by binding to IGF-1 as preadipocytes express more receptors for IGF-1 than for insulin [118]. Insulin binds to IGF-1 that results in the phosphorylation of cAMP response element-binding protein (CREB) through cAMP and phosphatidylinositol-3 kinase (PI3K) [119]. CREB is activated early to positively regulate the expression of CCAAT/enhancer binding protein alpha (C/EBP*α*) and peroxisome proliferator-activated receptor gamma (PPAR*γ*) [117]. Insulin also favors PPAR*γ* stimulation by inhibiting necdin that inhibits CREB stimulation of PPAR*γ* [117]. Through a serine/threonine protein kinase- (AKT/PKB-) mediated phosphorylation, insulin causes nuclear exclusion of forkhead transcription factor 1 (FOXO1) [120] and forkhead transcription factor 2 (FOXA2) that are both antiadipogenic transcription factors. GATA binding protein 2 (GATA2) is another target of AKT/PKB-mediated phosphorylation [121]. As these factors favor adipogenic differentiation of MSCs, MSCs become committed toward the adipogenic lineage. Following commitment, various transcription factors are activated that result in the adipogenic phenotype [122].

The treatment of MSCs with the above-mentioned induction factors results in an increased CREB phosphorylation that in turn transcriptionally activates C/EBP*β* [121]. The induction of C/EBP*β* leads to the activation of C/EBP*α* and Kruppel-like factor 5 (KLF5) [122], which in turn directly induce many adipocyte genes and specifically PPAR*γ* [123, 124]. PPAR*γ* is a key player in adipogenesis. It is not only necessary for adipogenesis [125] but also needed for maintaining the differentiated state [126]. After commitment, the expression of these factors differentiates committed MSCs to preadipocytes.

**Figure 3:**
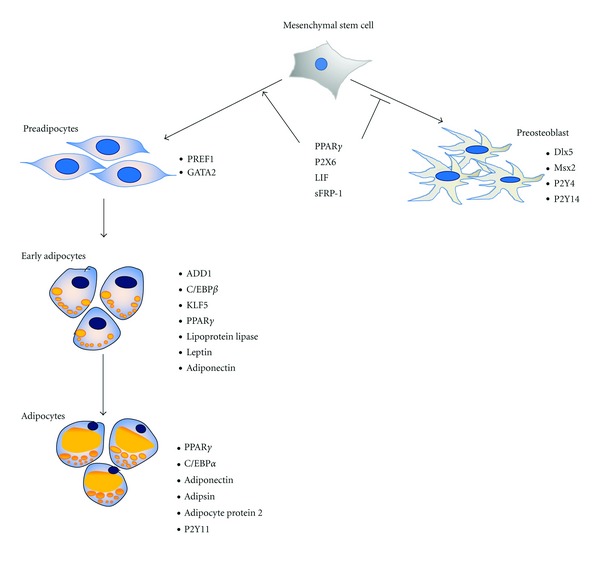
Differentiation of mesenchymal stem cells towards adipocytes. Peroxisome proliferator-activated receptor gamma (PPAR*γ*) commits MSCs towards adipogenic lineage. Preadipocyte factor 1 (Pref-1) and GATA binding protein 2 (Gata2) are expressed in preadipocyte. In early adipocyte determination and differentiation factor (ADD1), CCAAT/enhancer binding protein beta (C/EBP*β*), Kruppel-like factor 5 (KLF5), PPAR*γ*, lipoprotein lipase (LPL), leptin and adiponectin are expressed. The early adipocyte develops into the adipocyte that expresses PPAR*γ*, C/EBP*α* adiponectin, adipsin, adipocyte protein 2, and purinergic receptor P2Y 11 (P2Y11).

Preadipocytes are flat phase-dark spindle-shaped cells (see Figure 3). In culture, the appearance of these cells is similar to fibroblasts or smooth muscle cells. Preadipocyte factor 1 (Pref-1) is a transmembrane protein and is highly expressed in these cells. They also express Gata2 [127]. The expression of these markers is completely abolished in mature adipocytes [127, 128].

The preadipocytes are differentiated into early adipocytes. Early adipocytes become spherical in shape (see Figure 3). These cells express adipocyte determination and differentiation factor (ADD1), C/EBP*β*, KLF5, PPAR*γ*, lipoprotein lipase (LPL), leptin, and adiponectin [128] as key molecules.

Adipocytes are round in shape with large perilipin-coated lipid droplets that displace nuclei to the cell periphery (see Figure 3). These cells acquire cell arrest, sensitivity for insulin, and expression of adipokines. PPAR*γ*, C/EBP*α*, adiponectin, adipsin, adipocyte Protein 2 (aP2), and purinergic receptor P2Y, G-protein coupled, 11 (P2Y11) [35] are expressed by mature adipocytes.

### 2.6. Key Factors Controlling the Balance between Adipogenesis and Osteogenesis

**Figure 4:**
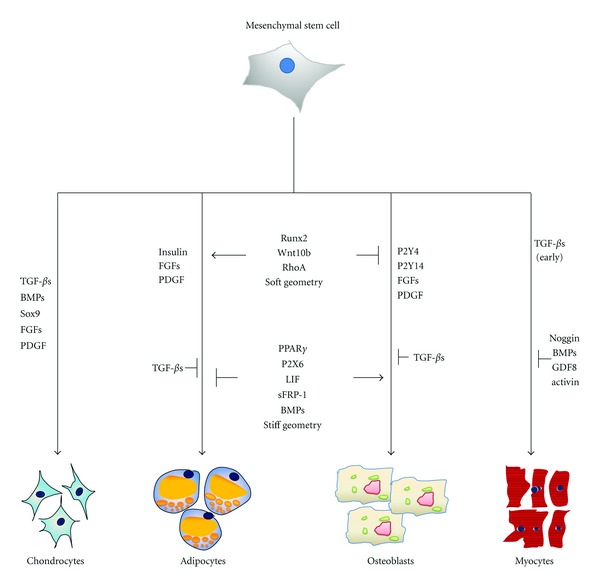
Key molecules regulating adipogenesis and osteogenesis in mesenchymal stem cells. Runx2, Wnt10b, RhoA, and soft geometry can induce osteogenesis while inhibiting adipogenesis. In contrast to this, PPAR*γ*, P2X6, LIF, sFRP-1, BMPs, as well as stiff geometry can trigger adipogenesis while inhibiting osteogenesis. Next to these key molecules, FGFs and PDGF can induce both adipogenesis and osteogenesis. Insulin can induce adipogenesis, while P2Y4 and P2Y14 can induce osteogenesis and TGF-*β*s can inhibit both adipogenic and osteogenic differentiation. The key molecules for the other major mesenchymal lineages are listed in the left-hand part of the figure for chondrogenesis and in the right-hand part for myogenesis. TGF-*β*s, BMPs, Sox9, FGFs, and PDGF are key triggers of chondrogenic differentiation. TGF-*β*s can induce early myogenesis, while Noggin, BMPs, GDF8, and activin can inhibit myogenic differentiation.

Summarizing the above-mentioned findings, some factors were found to be upregulated in adipogenesis but downregulated in osteogenesis or vice versa. These can be hypothesized to be key factors triggering differentiation into the adipogenic or osteogenic lineage (see Figure 4) [129]. An increasing expression of P2X6 was found during adipogenesis and a decreasing expression of the same factors in osteogenesis [35]. Leukemia inhibitor factor (LIF) and dexamethasone induce adipogenic differentiation and at the same time inhibit the maturation of osteoblasts [130, 131]. Similarly, some osteogenic differentiation triggering factors such as Runx2, Wnt10b, and bone morphogenetic proteins (BMPs) can inhibit adipocytes differentiation [132, 133]. Recently, secreted frizzled-related protein 1 (sFRP-1) was demonstrated to initiate adipogenesis and inhibit osteogenesis, and delta-like 1 (preadipocyte) factor 1 oppositely induces osteogenesis but inhibit adipogenesis through Wnt and NF-*κ*B signaling [134]. Constitutively active RhoA can induce hMSCs into osteoblasts; however, negatively expressed dominant-RhoA committed those MSCs to become adipocytes [135]. Transforming growth factors (TGF-*β*s) can trigger MSCs to commit towards myocytes and chondrocytes while inhibiting adipocyte, osteocyte, and endothelial cell differentiation [136, 137]. Fibroblast grow factors (FGFs) and platelet-derived growth factor (PDGF) are involved in the differentiation into adipogenic, osteogenic, and chondrogenic lineages [138]. Physico-mechanical features by extracellular matrix components can influence MSCs fate too. A soft local structural geometry can trigger adipogenic differentiation, while osteoblasts can be differentiated from MSCs in stiff scaffolds [76, 139, 140]. Cell-cell contact between MSCs also has an impact on their fate. Usually adipogenic differentiation requires larger number of cells than the initiation of osteogenic differentiation [35]. The MSCs microenvironment controls differentiation due to changes of cell shape and the cytoskeleton [139]. Finally, physicochemical factors such as oxygen tension, ionic strength, and pH can also mediate stem cell proliferation and differentiation [141–143].

## 3. MSCs in Clinical Trials

Human MSCs show potential for various therapeutic applications and have attracted the attention for clinical investigations. In addition to their multipotency, these cells secret immunosuppressive cytokines [144]. The low-immune characteristics make them a suitable candidate cell for allogenic therapeutic use, without stimulating the immune response in immunocompetent patients [145, 146]. The hMSCs are expected to repair damaged tissue or stimulate the damaged tissue through cytokines to regenerate themself. At present these cells are being investigated in many clinical studies at different phases to treat various diseases such as osteogenesis imperfecta, chronic and acute myocardial infarction, and graft versus host disease [147–149]. The public clinical trial database shows 123 studies investigating MSCs for a variety of therapeutic purposes [150]. The majorities of these studies are in phase I, phase II, or are a mixture of phase I and phase II [150].

Interestingly the clinical studies do not only address diseases to be expected to be treated by MSCs, but all kinds of diseases with cancers being the most prominent one.

At the moment, there are approximately 3000 studies with stem cells, and most of them seem to be effective due to indirect effects such as paracrine signaling or immunomodulatory effect of stimulating local progenitor cells to repair the damaged tissue. There can be no doubt that stem cells will be a key tool in regenerative medicine in the future.

## 4. Conclusion

Human MSCs will be an attractive source for regenerative medicine approaches in the future. They are already tested for new therapies for many diseases such as bone fracture repair, cartilage repair, and cardiovascular diseases. Other illnesses such as diabetes, stroke, multiple sclerosis, amyotrophic lateral sclerosis (ALS), and cancer are on the way as well. Inspiringly, more than 3000 clinical trials are performed for utilizing stem cells to treat above diseases at the moment. But astonishingly most of them, actually more than 2500 are for cancer (>2,500), thus not for an approach where repair is predominant, as could be expected from stem cell treatments. Most of these approaches are still in Phase I or Phase II and provide at least some new insight. However, it must be considered carefully why most of them have only short-term positive effects, and long-term benefits are missing for nearly all stem cell therapies at the moment. In order to solve this problem, more in-depth investigations are required.

Stem cells have paracrine effects on the neighboring progenitor and somatic cells, whose underlying mechanisms are still unclear. In addition, some of their beneficial properties seem to be due to immunomodulatory effects. This is an astonishing result because before, the expected effect of stem cells was the repair of damaged tissues or organs by differentiating into the cells whichever form and are functional in the targeted tissue or organ. This, of course, is still a main goal. However, how to stimulate and induce stem cells properly to achieve the desired differentiation and repair of the tissue obviously cannot be pinpointed to currently good enough. Therefore, key molecules which regulate and determine stem cell fate (some shown in Figure 4) are vital for addressing this question. In addition, the microenvironment of the cells seems to be equally important for improving stem cell therapies. Here a mimic of the stem cell niche *in vitro* is the objective. It might be necessary to fully induce stem cell differentiation for example, by modifying key molecule ligands or by providing the necessary physic-chemical environment to differentiate and stabilize the differentiated cells. For this, more work should be done to understand the underlying mechanisms of interaction between stem cells and their microenvironment. Last not least, in light of the reprogramming possibilities which have been discovered in the last five years, the plasticity of mesenchymal stem cell must be reinvestigated and reevaluated. Taken together, mesenchymal stem cells might give us possibilities for repair of damages tissues and organs in the future which we are just starting to discover.

## Figures and Tables

**Table 1:** Comparison of MSCs derived from different sources.

	Bone marrow	Adipose tissue	Other sources
Isolated methods	painful with invasive procedure	not additional pain; less invasive procedure	no pain; no invasive procedure from UCB, CB placenta
	100% success rate	100% success rate	63% success rate
Surface antigens or markers	CD106 MEST higher expression	CD49b, CD54, CD34; Ki-67, CDCA8, CCNB2 higher expression; chemokine receptors	CTGF, BMP antagonist 1 high expression in UC-MSCs
Differentiation potential	not restricted	not restricted	stronger osteogenic differentiation of UCB-MSCs; no adipogenic differentiation of UCB-MSCs
Proliferation	lowest	highest	high in CB-MSCs
Migration capacity	high	high	high in placenta-MSCs, low in UC-MSCs
Morphology	larger	normal	normal
Apoptosis tolerance	not high	high	not high

**Table 2:** Osteogenic differentiation factors.

Protocols	Reference
0.01 *μ*M 1,25-dihydroxyvitamin D3, 50 *μ*M ascorbate-2-phosphate, 10 mM *β*-glycerophosphate	[26, 113]
10 mM *β*-glycerophosphate, 0.1 mM ascorbic acid, 1 *μ*M dexamethasone and a pulsed electromagnetic field (PEMF)	[80]
50 *μ*g/mL ascorbic 2 phosphate, 10^−8 ^mM dexamethasone and 10 mM *β*-glycerol-phosphate and Matrigel	[92]
3D type I collagen matrices and 10% or 12% uniaxial cyclic tensile strain at 1 Hz for 4 h/day	[93]
Artificial extracellular matrices containing collagen and chondroitin sulfate with hydrostatic pressure (HP)	[81]

## Abbreviations

ADD1: Adipocyte determination and differentiation factor
AT: Adipose tissue
bFGF: Fibroblast growth factor
BM: Bone marrow
BMP: Bone morphogenetic protein
CCNB2: Cyclin B2
CDCA8: Cell division cycle associated 8
CREB: cAMP response element-binding protein
CTGF: Connective tissue growth factor
Dlx5: Distal-less homeobox 5
ECM: Extracellular matrix
EGF: Epidermal growth factor
ESCs: Embryonic stem cells
FGF: Fibroblast growth factor
FOXO: Forkhead transcription factor
GATA2: GATA binding protein 2
HP: Hydrostatic pressure
HSCs: Hematopoietic stem cells
iPS: Induced pluripotent stem cells
ISCT: International Society of Cellular Therapy
KLF5: Kruppel-like factor 5
LIF: Leukemia inhibitor factor
LPL: Lipoprotein lipase
MEST: Mesoderm-specific transcript homolog
MSCs: Mesenchymal stem cells
Msx2: Msh homeobox homologue 2
OPN: Osteopontin
Osx: Osterix
PAI-1: Plasminogen activator inhibitor-1
PB: Peripheral blood
PDGF: Platelet-derived growth factor
PEMF: Pulsed electromagnetic field
Pref-1: Preadipocyte factor 1
Runx2: Runt-related transcription factor 2
sFRP-1: Secreted frizzled-related protein 1
SM: Skeleton muscle
TGF-beta: Transforming growth factor beta
UC: Umbilical cord
UCB: Umbilical cord blood.
